# Modeling Spatio-Temporal Variations in the Habitat Utilization of Swordtip Squid (*Uroteuthis edulis*) in the East China Sea and Southern Yellow Sea

**DOI:** 10.3390/ani13223492

**Published:** 2023-11-12

**Authors:** Xiaodi Gao, Yazhou Jiang, Xingwei Yuan, Linlin Yang, Jianzhong Ling, Shengfa Li

**Affiliations:** 1 East China Sea Fisheries Research Institute, Chinese Academy of Fishery Sciences, Shanghai 200090, China; xd_gao@foxmail.com (X.G.);; 2 Key Laboratory of East China Sea Fishery Resources Exploitation, Ministry of Agriculture and Rural Affairs, Shanghai 200090, China

**Keywords:** *Uroteuthis edulis*, habitat suitability, species distribution models, environmental driver, seasonal variation

## Abstract

**Simple Summary:**

The swordtip squid (*Uroteuthis edulis*) is both of commercial and ecological importance and vital in the coastal China ecosystem. However, the limited ecological research requires further investigations into its habitat preferences. To address this, we studied how the habitat of *U. edulis* varies and what environmental factors drive its movements across different seasons in the East China Sea (ECS) and southern Yellow Sea. The study found that *U. edulis* predominantly inhabited the central and southern regions of the ECS, with a slight shift in the geometric center of its habitat across seasons. The preferences for sea surface temperature, sea surface height, and depth were the primary factors affecting its distribution. During summer and autumn, the suitable habitats of *U. edulis* were larger and expanded northwards towards the coastline, but in spring and winter, they retreated southwards to waters near the edge of the ECS continental shelf. These patterns are likely influenced by the changing mixture of ocean currents and varying environmental conditions throughout the year. This study provides valuable insight into how *U. edulis* is distributed in response to the changing environment, which can help to better manage and protect their populations.

**Abstract:**

Accurately modeling the distribution of keystone species is of utmost importance to gain a comprehensive understanding of their complex ecological dynamics and to develop effective strategies for sustainable scientific management. In the coastal China ecosystem, the swordtip squid (*Uroteuthis edulis*) stands out as a keystone species with significant commercial and ecological value. Despite its importance, research on the ecological dynamics of this species remains limited and requires further investigation. To investigate the spatial and temporal variability in the distribution of *U. edulis* and identify the key environmental drivers in the East China Sea (ECS) and southern Yellow Sea across different seasons, we generated ensemble models using oceanographic variables and fishery-independent scientific survey data collected from 2016 to 2018. Our results revealed that *U. edulis* predominantly inhabited the central and southern regions of the ECS throughout the year. The primary environmental variables driving its distribution varied by season, with the sea surface temperature being the most important in spring, sea surface height in summer and autumn, and depth in winter. During summer and autumn, the suitable habitats of *U. edulis* were found to be largest and extended northwards towards the coastline. However, they migrated southwards to the waters near the edge of the ECS continental shelf with smaller suitable areas in the spring and winter. These results suggested that *U. edulis* exhibited season-specific habitat preferences and responded to changing environmental conditions throughout the year. The observed seasonal distribution patterns were likely influenced by the fluctuating mixture of waters (ocean currents) from different sources, with varying physical and chemical characteristics throughout the year. Our study provides baseline data for comprehending the population dynamics of *U. edulis* and highlights the significance of considering species’ habitat preferences in a dynamic environment.

## 1. Introduction

Studying the distribution of a species is significant as it provides insights into its ecological niche, including its environmental tolerances and resource requirements [1]. By examining species across their entire geographic ranges, we can quantify their distributional possibilities against diverse community backgrounds. This understanding is vital for identifying ecologically significant areas, establishing marine protected areas, and making informed conservation management decisions, especially for keystone species within the ecosystem [2,3,4].

The swordtip squid, *Uroteuthis edulis*, thrives in the pelagic layers of shallow oceans; it is characterized by a short lifespan and high turnover rate [5]. This cephalopod is widely distributed in the northwestern Pacific Ocean, with an annual commercial catch of approximately 1.5 × 10^4^ tons in the East China Sea (ECS) [6,7]. Since the 1990s, the rapid development of the fishing industry led to a decline in traditional fish resources, resulting in cephalopods becoming key components of ECS trawling catches. Meanwhile, *U. edulis* has gradually overtaken the common Chinese cuttlefish (*Sepiella maindroni*) to become the most abundant species [7,8,9]. However, the substantial fishing pressure on *U. edulis* necessitates the implementation of conservation interventions to ensure the sustainability of both populations and ecosystems. This requires a comprehensive understanding of the species spatiotemporal distribution dynamics [10].

Species Distribution Models (SDMs) have become valuable tools for investigating species’ habitats [11,12]. By quantifying the relationship between species occurrence data (e.g., presence/absence, abundance) and environmental covariates, SDMs can describe habitat preferences of species and predict potential distributions within and beyond the study area or periods [11,13,14]. Currently, SDMs are extensively employed in the domains of biogeography, conservation biology, and ecology. They have been successfully used to predict potential and critical habitats for key species, mitigate the risk posed by invasive species, and forecast the potential impacts of climate change, including terrestrial, freshwater, and marine ecosystems [15,16,17,18,19,20,21,22]. 

SDMs incorporate various algorithmic models, such as traditional regression models (e.g., generalized linear model), classification models (e.g., Classification tree analysis model), and machine-learning models (e.g., Random Forest model) [23]. However, these single-algorithm models have varying applicability ranges and underlying assumptions. Improper utilization may impact the accuracy and reliability of model predictions. To address this limitation, the concept of an “ensemble model” has emerged. The ensemble models combine multiple optimal single-algorithm models, harnessing the strengths of different models and considering their collective predictions [17,24,25]. 

*Uroteuthis edulis* is a migratory species with high phenotypic plasticity. Changes in oceanic environmental and physicochemical conditions at different spatial and temporal scales significantly impact their physiological and biological characteristics [7]. They exhibit seasonal migration influenced by the Kuroshio Current, Taiwan Warm Current, and China coastal currents [26,27]. Currently, researchers have focused primarily on studying their growth, reproduction, migration, and population structure [7,27,28]. Despite their iconic status and potential to act as ecosystem indicators, the habitat dynamics of *U. edulis* are poorly understood. Although a few studies have preliminarily explored the habitat utilization of *U. edulis*, the limitations of the study area and the single modeling method may lead to large uncertainties in the results [25,29,30]. Additionally, fishery-dependent data are influenced by fishing activities, for which it is impractical to establish a species distribution map solely [31].

Thus, in this study, we utilized scientific survey data spanning three years to investigate the spatiotemporal distribution patterns of *U. edulis* in the ECS and southern Yellow Sea. Specifically, we constructed four ensemble models to identify dominant environmental drivers of species distributions and generate seasonally predictive maps, illustrating the variation in the likelihood of occurrence across the study area and seasons. Our study enhances the understanding of the spatial and temporal variability in the environmental preference of *U. edulis* and provides a valuable data foundation and theoretical framework for the effective management of this fishery.

## 2. Materials and Methods

### 2.1. Study Area and Sampling

**Figure 1:**
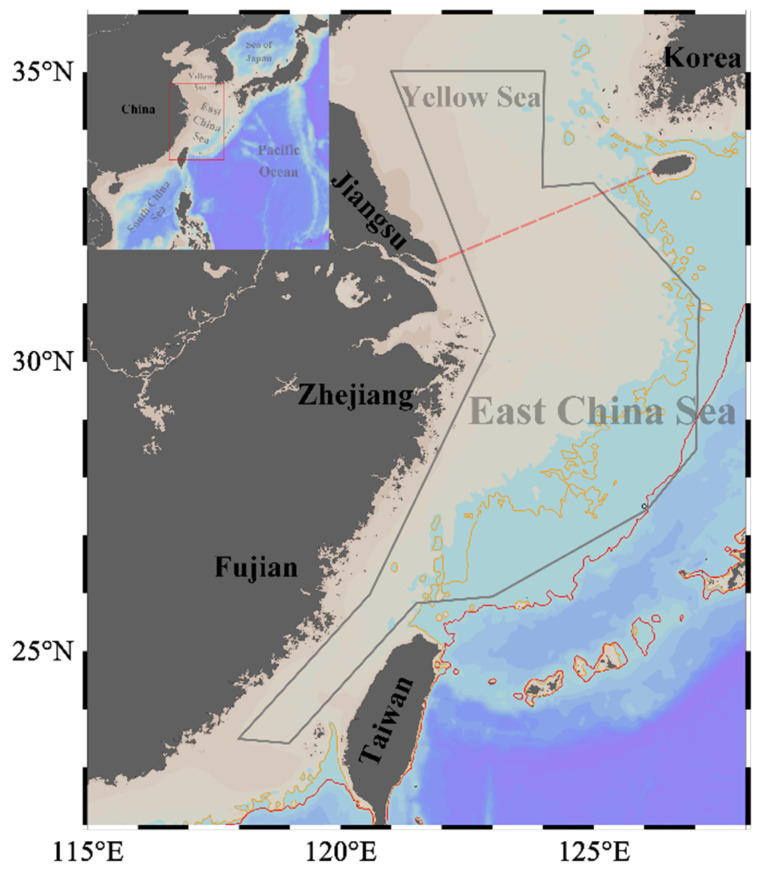
Study area in the East China Sea and southern Yellow Sea. The red box represents the study area along the coast of China. The grey convex hull outlines the survey edge. The yellow and red solid lines indicate the 100 m and 200 m isobaths, respectively. The red dashed line represents the boundary between the East China Sea and the Yellow Sea. Maps were generated using ETOPO1_2min bathymetry [32] in Ocean Data View 5.6.2 (http://odv.awi.de/, accessed on 30 May 2023) [33].

The biological samples were collected during the scientific demesal trawling surveys conducted between 2016 to 2018. The survey area extends from 118° E to 127° E and from 23° N to 35° N (Figure 1). This region, encompassing the continental shelf of the ECS (20–200 m in depth) and the southern waters of the Yellow Sea (20–80 m in depth), hosts a highly productive marine ecosystem that supports a diverse community of migratory pelagic species. This is due to the joint effect of seasonal upwelling, a western boundary current in the North Pacific Ocean, and the interactions among local surface winds, currents, and the complex geomorphology [22]. 

The surveys were carried out during each of the four seasons: spring (May), summer (August), autumn (November), and winter (January to February). Standard bottom-trawling gear with dimensions of 4 m × 100 mesh and a cod-end mesh size of 20 mm was used for 60 min at a constant speed of 2–3 knots, following the established sampling protocol for bottom trawling. All catches were identified and classified by species. The presence or absence data of *U. edulis* were recorded for each survey station. The number of survey stations varied slightly each year, depending on weather conditions or topographical roughness. Most of the surveys were conducted during the daytime, but considering the diel vertical migration of *U. edulis*, site data from nighttime surveys were not used in this study. As a result, the data from a varying number of survey stations for each season were kept to construct the models (Table S1). 

### 2.2. Environmental Variables

A total of fourteen environmental variables were initially selected by integrating the analysis of historical literature [29,30,34,35]. These variables consist of the sea surface temperature (SST), sea surface salinity (SAL), chlorophyll-a concentration (Chl), net primary production (nppv), total phytoplankton (phyc), dissolved oxygen concentration (O_2_), phosphate (po4), nitrate (no3), dissolved silicate (si), pH, sea surface height (SSH), mixed layer thickness (MLT), depth, and distance to the nearest coast. Oceanographic variables were obtained from the European Union Copernicus Marine Environmental Monitoring Service (CMEMS2) (https://marine.copernicus.eu/, accessed on 26 April 2023) with a spatial resolution of 0.25° × 0.25° and a monthly time resolution. To avoid the multicollinearity of environmental variables affecting the predictive ability of models, we conducted Pearson correlation matrix analysis and Variance Inflation Factors (VIFs) to test for all explanatory variables. Only environmental variables with a collinearity < 0.7 and VIF < 4 were retained [36,37]. These selected environmental variables were then used as explanatory variables in the models and matched with the distribution data of *U. edulis*.

### 2.3. Ensemble Model Construction

Following the selection of the final environmental variables, ten different SDM algorithms were developed and evaluated using the presence–absence data of *U. edulis* within the Biomod2 package (Table 1). This package constitutes a suite of statistical algorithms to compute the habitat suitability index (HSI), where the values closer to or equal to 1 represent potential habitat areas [38]. Considering the physiological tolerance to physical and abiotic environmental conditions of migratory species in different life stages [39], SDMs were constructed separately for the four seasons. Approximately 282–436 survey stations were selected in the study area for each season (Table S1). Each model was run 10 times, with 80% of the distribution data randomly selected as a training dataset and the remaining 20% as a validation dataset to evaluate model accuracy. All single models were initially assessed using the area under the receiver operating characteristic curve (AUC) [1,40]. The AUC ranges from 0 to 1, with values above 0.8 indicating good model accuracy. As a result, we obtained a total of 100 habitat model simulations for each season. To ensure the reliability of our ensemble models, we only kept the single models with a threshold of an AUC > 0.8. These selected models were then used to construct the ensemble model.

After evaluating the performance evaluation of single-algorithm models, weighted mean ensemble models were constructed for the squid habitat. These ensemble models were created by assessing the weights of the selected single SDMs based on their AUC values. The formula for single-model weighting is as follows:

(1) $$W_{j}=\frac{r_{j}}{\sum_{j=1}^{h}r_{j}}$$

where *W_j_* is the weight of the *j*th single model, *r_j_* represents the AUC value of the *j*th single model, and *h* is the number of models with an AUC > 0.8. 

The importance value, ranging from 0 to 1, was calculated for each environmental variable to identify the key factors that affect the habitat of *U. edulis* in different seasons. A higher value indicates a greater influence of the variable on the model, while a value of 0 implies no influence of that variable on the model. The higher the value, the more influence the variable has on the model. A value of 0 assumes no influence of that variable on the model. The above analyses were conducted using the Biomod2 package in R 4.0.3.

### 2.4. Suitable Habitat Area and Centroid Shifts

The seasonal habitat distribution of *U. edulis* was visualized by predicting the HSI for each spatial cell (0.25° × 0.25°) using the ensemble distribution model. The habitat was classified into four categories based on the HSI: unsuitable area (HSI < 0.2), low suitability area (0.2 ≤ HSI < 0.4), moderate suitability area (0.4 ≤ HSI < 0.6), and high suitability area (HSI ≥ 0.6) [41]. The total suitable habitat for *U. edulis* was determined as the sum of the low, moderate, and high suitability areas. The environmental preference of *U. edulis* in each season was further understood by characterizing the range of the most important environmental variables in the high suitability area.

Meanwhile, we calculated the seasonal longitudinal geometric center (*LONG*) and latitude geometric center (*LATG*) of the HSI to describe the centroid shifts in species distribution. The seasonal distribution centroid was determined as follows [42]:

(2) $$LONG=\frac{\sum \left(Longtitude_{\left(i,s\right)}\times HSI_{\left(i,s\right)}\right)}{\sum HSI_{\left(i,s\right)}}$$

(3) $$LATG=\frac{\sum \left(Latitude_{\left(i,s\right)}\times HSI_{\left(i,s\right)}\right)}{\sum HSI_{\left(i,\;\;s\right)}}$$

where *LONG* and *LATG* represent the longitude and latitude of the centroid of *U. edulis* distribution in season *s*, respectively. *Longitude*_(*i, s*)_ and *Latitude*_(*i, s*)_ are the longitude and latitude of the *i*th grid corresponding in season *s*. *HSI*_(*i, s*)_ represents the HSI value of the *i*th grid in season *s*. All the analyses were conducted using R 4.0.3.

## 3. Results

**Figure 2:**
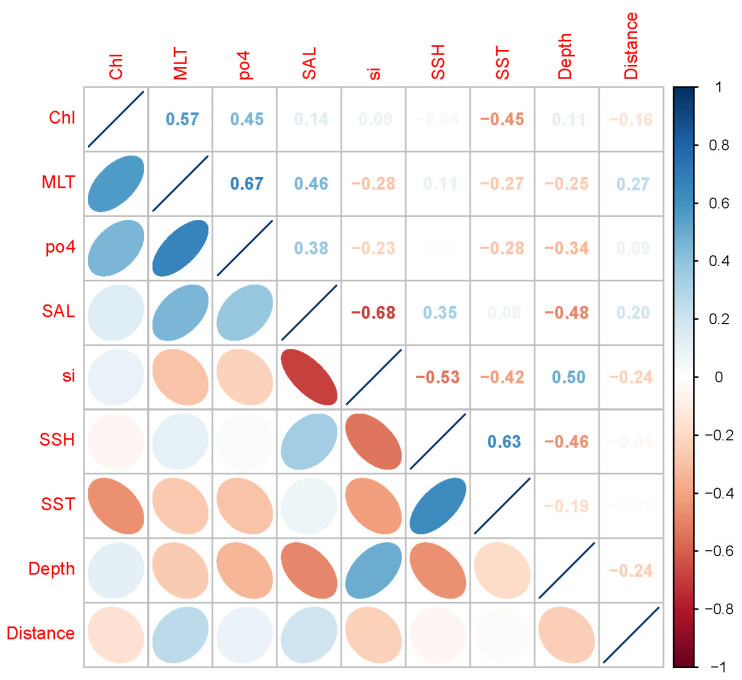
Pearson correlation coefficient between the selected environmental variables.

According to the Person correlation analysis (Figure 2 and Figure S1) and variance inflation factor screening (Table 2 and Table S2), a total of nine environmental variables (Chl, MLT, po4, SAL, si, SSH, SST, depth, and distance to the coast) were selected for modeling.

### 3.1. Model Accuracy Measures

**Figure 3:**
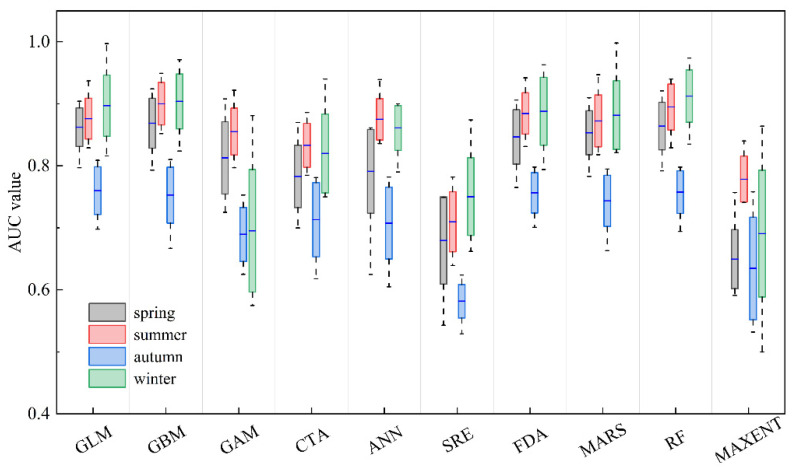
Boxplot of the evaluation metrics of ten single-algorithm models for four seasons, based on the area under the receiver operating characteristic curve (AUC). The blue line represents median values, and the boxes represent the interquartile range (rectangle). The whiskers represent the data range. The meanings of model codes are shown in Table 1.

The AUC values of the ten single models for the four seasons are presented in Figure 3. The ranges of AUC values for the single models in spring, summer, autumn, and winter were 0.543–0.924, 0.639–0.949, 0.529–0.840, and 0.500–0.998, respectively. The GLM and GBM models exhibited the best predictive performance across all four seasons. 

For each season, the single-algorithm models used in the ensemble models are displayed in Table 3. The AUC values of the ensemble models (AUC_c_) for all four seasons were greater than 0.8 and higher than the optimal models GLM and GBM, indicating that the ensemble models exhibited higher predictive accuracy in predicting the habitat distribution of *U. edulis* compared to the single-algorithm models. 

### 3.2. Key Environmental Variables and Suitable Environmental Ranges

**Figure 4:**
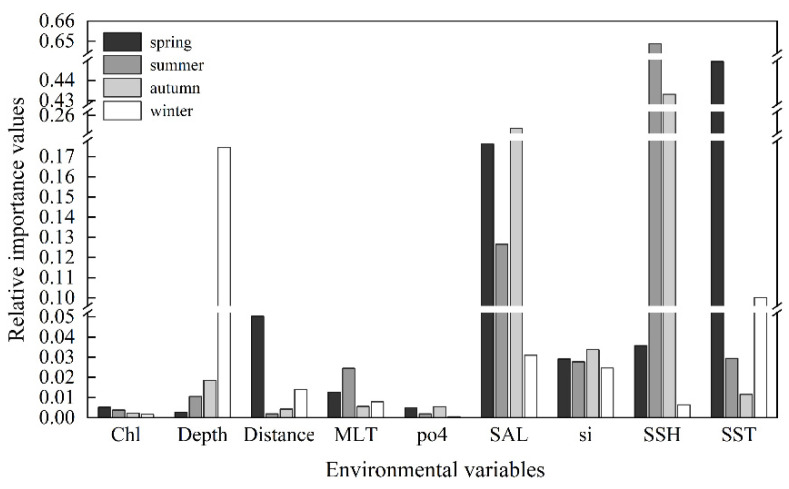
The relative importance of environmental variables to the habitat suitability of *Uroteuthis edulis* in each season. The abbreviations of environmental variables are defined in Table 2.

On average, the three environmental factors with the highest importance value were SSH (0.282), SST (0.147), and SAL (0.144). However, the most influential variable varied with the seasons (Figure 4). SST had the greatest impact on the species distribution in spring, with an importance value of 0.445. The preferred SST range for spring was 18.4 °C to 25.6 °C, while for summer and autumn, it was relatively higher, ranging from 26.4 °C to 29.5 °C and 25.6 °C to 27.7 °C, respectively. In winter, the preferred SST was the lowest, ranging from 17.4 °C to 23.6 °C.

SSH emerged as the most influential environmental variable impacting the distribution of *U. edulis* during summer and autumn, with importance values of 0.649 and 0.433, respectively. The preferred range of SSH for spring was 0.37 m to 0.59 m, while for summer and autumn, it peaked at 0.37 m to 0.63 m and 0.43 m to 0.70 m, respectively. In winter, the preferred SSH range was 0.36 m to 0.62 m.

Depth was the most important environmental variable affecting the distribution of *U. edulis* in winter, with an importance value of 0.174. The preferred depth ranges for spring, summer, and autumn consistently fell between 19 m and 229 m, while the preferred depth during winter ranged from 87 m to 229 m.

SAL was ranked as the second most crucial variable in all seasons except winter, underscoring its pivotal role in shaping the *U. edulis* habitat. The range of SAL variations across different seasons was relatively narrow, ranging from 32.4 to 34.6, indicating a small degree of variation. The preferred environmental variables for the habitat of *U. edulis* in different seasons are summarized in Table 4.

### 3.3. Spatial Patterns of Habitat Suitability and Centroid Migration Routes

**Figure 5:**
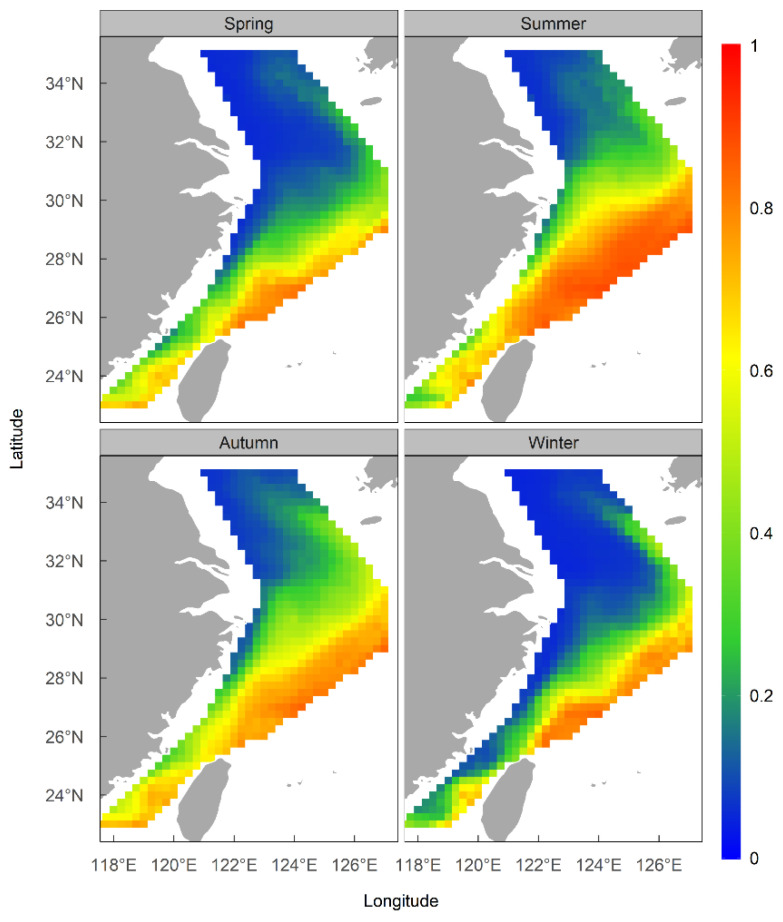
Habitat suitability of *Uroteuthis edulis* in the East China Sea and Southern Yellow Sea in each season using the ensemble models.

**Figure 6:**
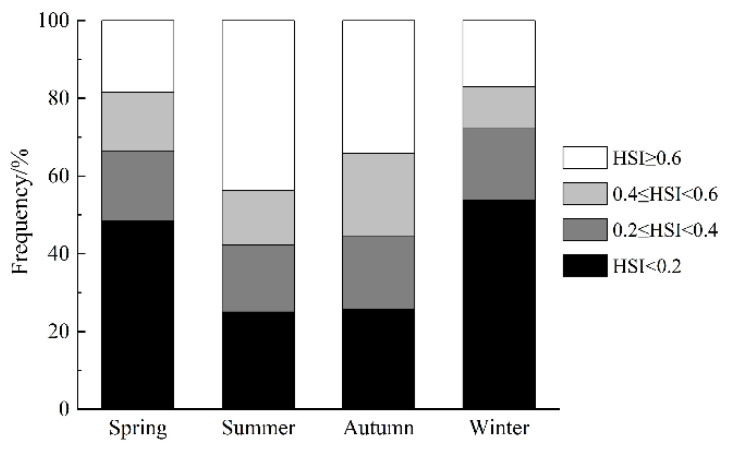
Habitat suitability index (HSI) of *Uroteuthis edulis* in the East China Sea and southern Yellow Sea in each season.

The results of the ensemble model prediction revealed that *U. edulis* was primarily distributed in the southeastern of the ECS, ranging from 119° E to 127° E and from 24° N to 29° N, across all four seasons. However, there were variations in the extent and centroid of the suitable habitat (Figure 5 and Figure 6). The largest total suitable habitat area was observed in summer, followed by autumn, with areas of 32.99 × 10^4^ km^2^ and 32.62 × 10^4^ km^2^, respectively. Additionally, the proportion of high suitability habitats (HSI > 0.6) was the highest in summer (43.78%), with the largest suitable habitat area of 19.44 × 10^4^ km^2^. In contrast, the HSI was lowest in winter, with 53.76% of the area having an HSI below 0.2. The total suitable habitat area in winter was the smallest, measuring only 20.47 × 10^4^ km^2^ (Figure 4, Table 5). 

**Figure 7:**
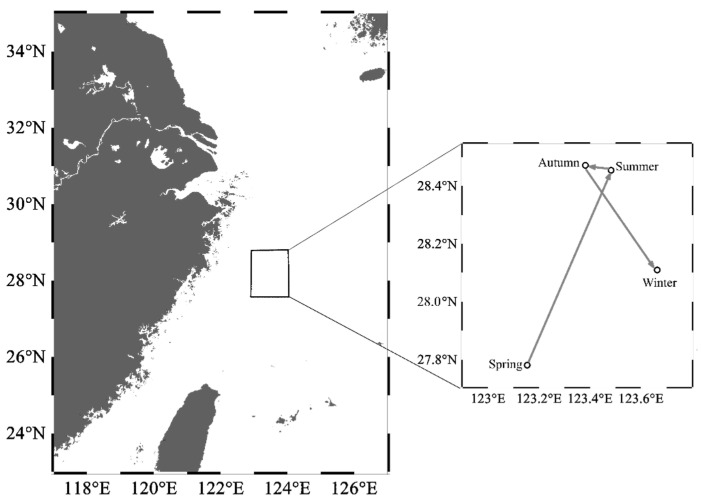
Seasonal centroid migration routes of *Uroteuthis edulis* in the East China Sea and southern Yellow Sea. Maps were generated using Ocean Data View 5.6.2 [33].

From spring to winter, the geometric center of the HSI exhibited a trend of displacement, initially towards the northeast and subsequently towards the southeast direction (Figure 7). In spring, the HSI geometric center was located near 123° E, 27.5° N–28° N waters. In summer, the geometric center moved 0.33° eastward in longitude and 0.67° northward in latitude. The latitudinal geometric center in autumn was similar to that in summer, but the longitudinal geometric center shifted westward by 0.10°. In winter, the geometric center continued to shift eastward by 0.28°, near 124° E waters, while moving southward by 0.36° in terms of latitude, which was near 28° N waters. 

The findings indicate that the oceanographic conditions in the study area were more suitable for *U. edulis* during summer and autumn compared to spring and winter. Although there was only a slight change in the geometric center of the HSI across seasons, the variation in suitable habitat areas demonstrated a clear seasonal pattern of movement for the key habitat of *U. edulis*. This pattern involved the habitat shifting back and forth between the southeastern offshore waters of the study area and more distant offshore waters, as well as migrating between the southern and northern regions.

## 4. Discussion

In this study, we employed ensemble models to integrate distribution data and environmental variables from the ECS and the southern Yellow Sea to elucidate the seasonal habitat utilization of *U. edulis* and identify the key environmental factors influencing their distribution. Our findings indicated that *U. edulis* exhibits distinct seasonal preferences in its habitat use, which can be attributed to its adaptative capacity to cope with key environmental conditions throughout the year. 

### 4.1. Environmental Preference

Cephalopods, as a species with high phenotypic plasticity and a short life cycle, exhibit a strong sensitivity to the environment, making their spatial distribution highly influenced by environmental factors [43,44]. During their ontogenesis, cephalopods appear to actively seek out optimal environmental conditions [45]. In our study, SSH, SST, SAL, and depth seemed to be the key environmental variables that determine the distribution of *U. edulis* in the ECS and the southern Yellow Sea.

SST and SAL play crucial roles in influencing a large range of biological processes, such as growth, reproduction, larval development, and the recruitment of organisms [46,47,48]. Consequently, they drive the spatial distribution of species at a macroecological level. SSH, on the other hand, is generally associated with heat flux, wind, and eddy currents, which impact the transport of marine matter and indirectly indicate the level of primary productivity [49]. Additionally, depth directly correlates with the watercolor, transparency, water flow direction, dissolved oxygen levels, and food availability [50]. It is worth noting that the relative importance of these environmental factors varies with seasons, suggesting that *U. edulis* may exhibit different environmental tolerances at different life history stages and under different environmental conditions [30,39,51,52].

In spring, SST was found to be the most influential factor in determining the distribution of *U. edulis*. Spring is the peak period for the spawning and hatching of *U. edulis*. The water temperature during this time plays a crucial role in determining the timing and success rate of egg hatching, as well as the growth, migration, and distribution of the young [7,39]. Based on the biological data we collected, the catch composition during this period predominantly consisted of juveniles, with an average mantle length of 59.2 mm. This indicates the importance of understanding the specific environmental conditions, particularly SST, that are favorable for the successful reproduction and early life stages of *U. edulis* during spring.

SSH emerged as the most key environmental factor during the summer and autumn. This could be attributed to the presence of strong eddies during these periods, which create favorable conditions for *U. edulis* and make it easy to form fishing grounds [28]. The suitable SSH values for the *U. edulis* habitat exhibited a high value during summer and autumn, while a low value was observed during spring and winter. This pattern aligns with the annual variation amplitude of SSH in the area, indicating a positive relationship between the water’s SSH trend and the seasonal SSH preference of *U. edulis* [53].

Depth was the most influential factor in the winter. As the temperature decreases, the main stream of the Kuroshio warm current narrows, while the coastal cold-water mass strengthens [30]. Consequently, *U. edulis* migrates southward along the Kuroshio to the marginal waters of the continental shelf for wintering. Additionally, the need for food could serve as another driving factor behind the migration of *U. edulis* to deeper waters during winter. Guo et al. (2023) speculated that *U. edulis* feeds in deeper water layers during this season, based on a comparison of the fatty acid composition between autumn and winter [54]. Besides, the vertical migration to the spawning depth for spawning preparation may also be a contributing factor [48]. The migration behavior of *U. edulis* in response to changes in temperature, feeding, or spawning behavior underscores the importance of comprehending the role of depth in shaping the winter distribution patterns of *U. edulis*.

SAL was also identified as one of the key variables [44]. The study revealed that *U. edulis* preferably inhabited waters with high salinity levels (32.8–34.6), which aligned with the findings of previous research conducted by Fang (1994) and Chen et al. (2021) [29,55]. The suitable salinity range for *U. edulis* was characterized as being low in summer and high in winter, mirroring the seasonal variation observed in surface water salinity in the ECS [56,57]. This correspondence indirectly validates the high reliability of the predictive results obtained from the ensemble models.

### 4.2. Seasonal Variations in Habitat Utilization

Our findings aligned with previous studies that have observed that warm-water species, *U. edulis*, mainly inhabited south of 30°30′ N, with fishing grounds typically located between 121° E–126° E and 25° N–29° N, on the continental shelf of the ECS, at water depths ranging from 60 m to 200 m [27,28,58]. This particular region is the convergence zone of the Kuroshio Current and its branch, the Taiwan Strait Water, as well as the Mainland China Coastal Cold Current. The interactions among local surface winds, currents, and complex geomorphology give rise to seasonal stratified wind-driven upwelling and cyclonic cold eddies [59]. Consequently, this area exhibits high primary productivity and biodiversity, making it a crucial habitat and spawning ground for numerous marine organisms [60,61]. 

The distribution of *U. edulis* has been generally considered to be influenced by the changes in the cold water, the Taiwan Warm Current, and the extension or retreat of the main Kuroshio Current [28]. *U. edulis* tends to concentrate its distribution on the side of the warm water mass near the intersection of cold and warm water. Liao et al. (2006) pointed out that the spatio-temporal distribution of *U. edulis* fishing grounds is primarily associated with seasonal changes in thermal fronts and eddies [28]. Along the moving Kuroshio front, it provides the optimal sea temperature range for *U. edulis* [62]. Additionally, the cold eddies generated by the upwelling of subsurface water from the Kuroshio Current bring abundant nutrients and aggregate prey organisms, which are also beneficial for the aggregation of *U. edulis* [27,55,59,63].

The seasonal migration patterns of *U. edulis* involving movements between nearshore and offshore waters, as well as between south and north waters, are primarily influenced by the strength of the Kuroshio Current. In spring, the invasion of the Kuroshio Current is relatively strong, which affects the southward movement of *U. edulis*. They mainly concentrate near the upwelling area in the northeast of Taiwan. In summer, the suitable habitat for *U. edulis* extends northeastward, driven by the expansion of the front formed by the intersection of the Kuroshio Current and the coastal current of the continent. As the front moves, the suitable habitat shifts closer to the nutrient-rich nearshore areas [28,30]. In autumn and winter, the intensity of the Kuroshio Current weakens, causing *U. edulis* to gradually retreat southeastward towards the upwelling area near the northeast of Taiwan [30]. Therefore, in spring and winter, *U. edulis* mainly concentrated in the Wentai, Wenwai, and Yuwai fishing grounds near the Kuroshio Current and Taiwan Warm Current. In summer and autumn, their suitable habitats moved northward and extended towards the nearshore areas, reaching the Zhoushan, Zhouwai, and Yushan fishing grounds (Figure 3) [29,64]. 

The seasonal variations in suitable habitat areas could be attributed to both specific environmental preferences (such as temperature) and the population size of *U. edulis*. During cold seasons, the northeast monsoon intensifies, prompting *U. edulis* to migrate southward in search of a warmer environment [27]. Thus, *U. edulis* primarily congregates near the Kuroshio Current, which is renowned for its high temperature and salinity, leading to a smaller suitable habitat in the winter and spring. As temperatures rise, the distribution of *U. edulis* expands northward and moves closer to the coast, resulting in a larger suitable habitat. The increase in population size was another possible reason for the habitat expansion. Summer and autumn are the peak hatching periods for *U. edulis*, with the largest population size occurring in summer, followed by autumn [7,58]. Additionally, a study combining otolith microchemistry and water temperature information inferred that a summer spawning ground of *U. edulis* existed near the coastal waters of Zhoushan [51]. This finding may explain the expansion of the suitable habitat for *U. edulis* towards the coast in the summer. 

The Chinese government implements an annual summer fishing moratorium system from May to September each year. The seasonal variations in habitat utilization observed in our study provided evidence that this program effectively provides a vital period of rest for the *U. edulis* population, which significantly contributes to the improved protection and conservation of this valuable fishery resource [65,66].

### 4.3. Optimization of Species Distribution Models and Sustainable Utilization of Resources

Changes in habitats will lead to the redistribution of resource centers, which ultimately impacts the ecosystem’s service functions through trophic cascades. Consequently, it is crucial to comprehend the dynamics of fishery space [67]. The ensemble models, formed by combining the well-performing single models, exhibit a more robust predictive ability and have been proven to be beneficial for accurately predicting the habitat distribution of migratory cephalopods in the ocean [44]. The ensemble models can also account for habitat changes caused by variations in the importance of environmental variables across different single models [24,25,44]. Additionally, the fishery-independent scientific survey data used in this study provide a more accurate reflection of the spatial distribution of the species compared to fishery-dependent data [31,67].

The variations in organism tolerance to the environment at different life history stages ought to be taken into account when an SDM is constructed. Cephalopods exhibit year-round spawning with multiple peaks, leading to distinct population structures in different seasons. These different cohorts possess varied biological characteristics and distribution patterns and may also exhibit varying degrees of environmental tolerance [68]. This study not only showcased the superiority of ensemble modeling methods over single-modeling methods but also emphasized that the habitat model established by seasons (Table 2, $\bar{\mathrm{AUC}}$= 0.942) outperformed the overall model (AUC = 0.917). Therefore, it is necessary to consider species’ physiological characteristics and select an appropriate method when modeling the habitat dynamics of species.

Cephalopods are annual biological resources that have different spawning populations and generations coexisting in various seasons [68]. However, their dynamic habitat may not be effectively protected by static ocean management tools due to their high mobility [51,56,69]. Additionally, the recruitment of the population relies on the hatching of fertilized eggs and the survival of juveniles. Therefore, it is necessary to comprehend the spatio-temporal distribution of spawning groups and juveniles, precisely delineate the dynamic habitat of different spawning populations, and implement effective resource conservation for sustainable development. Furthermore, the potential impact of long-term climate warming on the habitat distribution of *U. edulis* must be considered. Monitoring population dynamics, particularly during the spawning peaks of *U. edulis* in March to May and October to November, is essential. It is also of utmost importance to protect spawning populations and juveniles, understand potential habitat loss and gain in long-term variations, in addition to establishing dynamic conservation areas. 

## 5. Conclusions

In summary, our findings demonstrated the seasonal environment preferences of *U. edulis*, emphasizing the importance of considering its varying environmental requirements at different life stages when studying the impacts of changing oceanographic conditions. Additionally, our study adds to the growing body of evidence supporting the superiority of ensemble models in predicting the habitat distribution of *U. edulis*, which provides a scientific foundation for establishing its spatial distribution pattern, predicting fishing grounds, and evaluating its response to climate change. Although we identified a seasonal migratory distribution pattern for *U. edulis*, it is important to note that our survey data did not adequately cover the core habitat of the species in either season. In the analysis, we also did not differentiate among different cohorts, which may have distinct growth and spawning strategies, potentially resulting in varying habitat suitability. These limitations may hinder our understanding of the spatial and temporal distribution patterns and resource assessment of the *U. edulis* population. Nonetheless, this study provides valuable insights into the conservation and sustainable management of cephalopod resources in the face of declining offshore fishery resources, significant changes in the catch structure, and increased fishing pressure on cephalopods. Future studies should aim to improve the spatial and temporal scale of habitat suitability modeling and understand distribution dynamics at all phases in the life cycle of *U. edulis*. 

## Figures and Tables

**Table 1:** Ten model algorithms used in the study.

Type	Algorithm	Code
Traditional regression model	Generalized additive model	GAM
	Generalized linear model	GLM
	Multivariate adaptive regression splines model	MARS
Classification model	Generalized boosted model	GBM
	Classification tree analysis model	CTA
	Flexible discriminant analysis model	FDA
Machine-learning model	Artificial neural networks model	ANN
	Random forest model	RF
	Maximum entropy model	MaxEnt
	Surface range envelope model	SRE

**Table 2:** Collinearity of the selected variables by calculating the Variance Inflation Factor (VIF).

Environmental Variable	Code	Units	VIF
Chlorophyll-a concentration	Chl	mg/m^3^	2.35
Mixed layer thickness	MLT	m	2.93
Sea surface salinity	SAL	psu	2.36
Dissolved Silicate	si	mmol/m^3^	2.76
Phosphate	po4	mmol/m^3^	2.17
Sea surface height	SSH	m	2.72
Sea surface temperature	SST	℃	2.82
Depth	Depth	m	1.91
Distance to the coast	Distance	×10^3^ km	1.38

**Table 3:** Model compositions and evaluation metrics for the ensemble models (AUC_c_) for each season.

Season	Number of the Models	Model Composition (AUC > 0.8)	AUC_c_
Spring	8	ANN, CTA, FDA, GAM, GBM, GLM, MARS, RF	0.950
Summer	9	ANN, CTA, FDA, GAM, GBM, GLM, MARS, MaxEnt, RF	0.951
Autumn	2	GBM, GLM	0.892
Winter	10	ANN, CTA, FDA, GAM, GBM, GLM, MARS, MaxEnt, RF, SRE	0.974

The means of model code are shown in Table 1.

**Table 4:** The optimal range for key environmental variables of the highly suitable habitat of *Uroteuthis edulis* in each season based on the ensemble models.

Season	Depth	SAL	SSH	SST
spring	19–229	33.3–34.5	0.37–0.59	**18.4–25.6**
summer	19–229	32.4–33.9	**0.37–0.63**	26.4–29.5
autumn	19–229	32.8–34.0	**0.43–0.70**	25.6–27.7
winter	**87–229**	33.7–34.6	0.36–0.62	17.4–23.6

The values of the most important environmental variables for each season are in bold.

**Table 5:** The habitat area of *Uroteuthis edulis* in the East China Sea and southern Yellow Sea at different levels in each season (units: ×10^4^ km^2^).

Season	Unsuitable Habitat	Suitable Habitat			
		Low	Moderate	High	Total
spring	20.62	7.88	6.70	8.23	22.85
summer	10.48	7.41	6.14	19.44	32.99
autumn	10.85	7.99	9.43	15.20	32.62
winter	23.00	8.12	4.77	7.81	20.47

## Data Availability

The data presented in this study are available on reasonable request from the corresponding author.

## Supplementary Materials

The following supporting information can be downloaded at: https://www.mdpi.com/article/10.3390/ani13223492/s1, Table S1: The number of survey stations with *Uroteuthis edulis* present (P) and absent (A) for each year. Figure S1: Pearson correlation coefficients among the fourteen variables; Table S2: Collinearity of the fourteen variables based on a calculation of the Variance Inflation Factor (VIF).
