# Supplementary material for: Modeling Spatio-Temporal Variations in the Habitat Utilization of Swordtip Squid (Uroteuthis edulis) in the East China Sea and Southern Yellow Sea
Source: Animals (Basel). 2023 Nov 12;13(22):3492. doi: 10.3390/ani13223492 (PMC10668723; doi:10.3390/ani13223492)
Supplement: Supplementary file 1 [file animals-13-03492-s001.zip › animals-2653116-supplementary.pdf]

Modeling Spatio-Temporal Variations in the Habitat Utilization of Swordtip Squid (*Uroteuthis Edulis*) in the East China Sea and Southern Yellow Sea

Xiaodi Gao [1,2], Yazhou Jiang [1,2], Xingwei Yuan [1,2], Linlin Yang [1,2], Jianzhong Ling [1,2], Shengfa Li [1,2,*]

[1]East China Sea Fisheries Research Institute, Chinese Academy of Fishery Sciences, Shanghai 200090, China;

[2]Key Laboratory of East China Sea Fishery Resources Exploitation, Ministry of Agriculture and Rural Affairs, Shanghai 200090, China

* Correspondence: lisf@ecsf.ac.cn

Table S1 The number of survey stations with *Uroteuthis edulis* present (P) and absent (A) for each year (Total$_y$) and each season (Total$_s$).

Year	Total$_y$	Spring		Summer		Autumn		Winter	
		P	A	P	A	P	A	P	A
2016	556	42	113	63	107	41	100	21	69
2017	540	52	104	74	70	33	107	23	77
2018	520	26	99	66	95	45	97	33	59
Total$_s$		423		436		475		282	

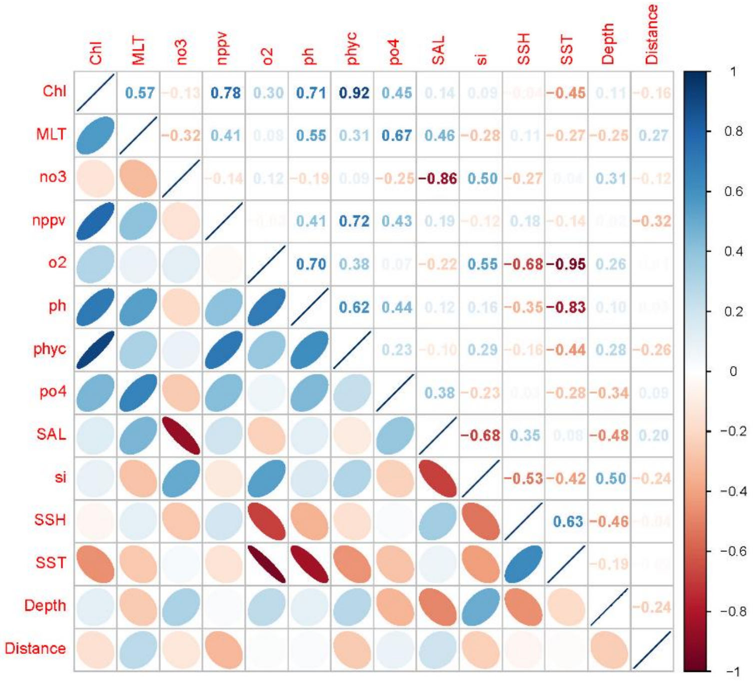

Figure S1 Pearson correlation coefficient among the fourteen environmental variables.

Table S2 Collinearity of the fourteen variables based on a calculation of the Variance Inflation Factor (VIF).

**Environmental variable**	**Code**	**Units**	**VIF**
Chlorophyll-a concentration	Chl	mg/m^3	27.22
Mixed layer thickness	MLT	m	4.20
Nitrate	no3	$mmol/m^3$	6.51
Total Primary Production of Phytoplankton	nppv	$mmol \times m^{-3}/d$	4.12
Oxygen concentration	O_2	$mmol/m^3$	32.97
pH	pH		8.30
Total Phytoplankton	phyc	$mmol/m^3$	21.22
Phosphate	po4	$mmol/m^3$	2.84
Sea surface salinity	SSS	psu	8.77
Dissolved Silicate	si	$mmol/m^3$	3.55
Sea surface height	SSH	m	3.01
Sea surface temperature	SST	℃	43.46
Depth	Depth	m	2.02
Distance to the coast	Distance	$\times 10^3$ km	1.54
